# Electronic Medication Monitoring-Informed Counseling to Improve Adherence to Combination Anti-Retroviral Therapy and Virologic Treatment Outcomes: A Meta-Analysis

**DOI:** 10.3389/fpubh.2015.00139

**Published:** 2015-05-19

**Authors:** Nienke Langebeek, Pythia Nieuwkerk

**Affiliations:** ^1^Department of Internal Medicine, Rijnstate Hospital, Arnhem, Netherlands; ^2^Department of Medical Psychology, Academic Medical Center, Amsterdam, Netherlands

**Keywords:** adherence, compliance, HIV infection, anti-retroviral therapy, meta-analysis

## Abstract

**Background:**

Adherence to combination anti-retroviral therapy for HIV infection is a primary determinant of treatment success, but is often suboptimal. Previous studies have suggested that electronic medication monitoring-informed counseling is among the most effective adherence intervention components. Our objective was to review available evidence about the effectiveness of monitoring-informed counseling and to aggregate findings into quantitative estimates of the effect of such intervention on medication adherence and virologic treatment outcomes.

**Methods:**

We searched PubMed for papers reporting on randomized controlled trials comparing intervention groups receiving monitoring-informed counseling as one of the intervention components versus control groups not receiving such counseling for their effect on medication adherence and viral load concentrations. The standardized mean difference (SMD) in adherence and the odds ratio (OR) of undetectable HIV RNA in intervention versus control groups were the common effect sizes. Random-effect models with inverse variance weights were used to aggregate findings into pooled effect estimates with 95% confidence limits (CI).

**Results:**

A total of 13 studies were included. Adherence was significantly higher in intervention groups than in control groups (SMD 0.51, 95% CI 0.31–0.71). Patients in intervention groups were significantly more likely to have undetectable HIV RNA concentrations than patients in control groups (OR 1.35, 95% CI 1.12–1.63). However, in studies in which monitoring-informed counseling was the only intervention component, the difference in adherence and virologic response between intervention and control groups was not statistically significant.

**Conclusion:**

Electronic monitoring-informed counseling improved adherence and virologic response compared with control groups not receiving such counseling in studies in which it was one out of multiple intervention components, but not in studies where it was the only intervention component.

## Introduction

Adherence to combination anti-retroviral therapy (cART) is a primary determinant of anti-retroviral treatment success. Sufficiently high levels of adherence to cART are necessary to achieve and sustain viral suppression and to prevent disease progression and death (1–3). Yet, many HIV-infected patients do not succeed in achieving or maintaining adequately high levels of adherence to cART (4). Adherence to cART is potentially amenable to intervention. Since the advent of cART, numerous interventions aimed at enhancing adherence to cART have been developed and evaluated. Several systematic reviews have reviewed and synthesized the effectiveness of such interventions to improve adherence and virologic treatment outcomes (5–8). Overall, these reviews have shown that various types of interventions can significantly increase adherence, but effects vary considerably across studies and most types of interventions have also been found not to produce significant effects in other studies.

An appreciable number of adherence-enhancing intervention studies have provided patients and/or their health care providers with objective information about the patients’ medication-taking behavior as one of the intervention components. In these studies, medication adherence is typically measured using an electronic medication monitoring device. Electronic medication monitoring devices register the time and date of each opening of the device, which is assumed to represent medication ingestion. The date and time of openings of the device over a long-time period can be shown to patients in the form of a graphical display. Such graphical feedback could make medication-taking behavior and the occurrence of non-adherence more concrete or real to the patient who may be unaware of suboptimal adherence. Personalized feedback based on the pattern of medication use could open discussions between patients and health care providers about adherence barriers and potential solutions to deal with these.

Research conducted across medical conditions have suggested that feedback on adherence performance and the accompanying counseling informed by recent adherence performance are among the intervention components that improve adherence most consistently (9–11). However, studies investigating the effectiveness of such monitoring-informed counseling among patients with chronic HIV infection have yielded inconsistent results. Some studies (12) have found significantly improved adherence and virologic treatment outcomes whereas others have found no beneficial effects (13).

Our objective was to review available evidence about the effectiveness of monitoring-informed counseling among patients who are prescribed cART for a chronic HIV infection and to aggregate findings into quantitative estimates of the effect of such intervention on medication adherence and virologic treatment outcomes. Moreover, we aim to identify study design features that are associated with stronger intervention effects.

## Materials and Methods

### Literature search

We searched PubMed for papers published from August 1996 to October 2014 using the following strategy:

((“intervention” [tiab]) OR [“intervention” [tw])] AND (HAART[title/abstract] OR CART[title/abstract] OR ART[title/abstract] OR ARV[title/abstract] OR ARVs [title/abstract] OR antiretroviral[title/abstract] OR anti-retroviral[title/abstract] OR anti-viral[title/abstract] OR antiviral[title/abstract] OR “Antiretroviral Therapy, Highly Active”[Mesh] OR “Anti-Retroviral Agents”[Mesh])) OR ((HIV Infections[MeSH] OR HIV[MeSH] OR hiv[title/abstract] OR hiv-1[title/abstract] OR hiv-2*[title/abstract] OR hiv-1[title/abstract] OR hiv2[title/abstract] OR hiv infect*[title/abstract] OR human immunodeficiency virus[title/abstract] OR human immune deficiency virus[title/abstract] OR human immuno-deficiency virus[title/abstract] OR human immune-deficiency virus[title/abstract] OR [(human immun*) AND (deficiency virus[title/abstract])] OR acquired immunodeficiency syndromes [title/abstract] OR acquired immune deficiency syndrome[title/abstract] OR acquired immuno-deficiency syndrome[title/abstract] OR acquired immune-deficiency syndrome[title/abstract] OR ((acquired immun*) AND (deficiency syndrome [title/abstract])) or “sexually transmitted diseases, viral”[mh]) OR HIV[title/abstract] OR HIV/AIDS [title/abstract] OR HIV-infected[title/abstract] OR HIV[title] OR HIV/AIDS[title] OR HIV-infected [title])) AND (adhere*[tiab] OR complian*[tiab] OR adhere*[tw] OR complian*[tw] OR Patient Compliance[MeSH] OR Medication Adherence [MeSH])) AND (“1996/01/01”[PDat]: “2014/12/31”[PDat])))

The reference lists of the papers retrieved were reviewed for additional relevant publications. Additionally, we searched abstracts from the International AIDS conference (years 2006, 2008, 2010, 2012, 2014), the IAS Conference on HIV Pathogenesis, Treatment, and Prevention (years 2007, 2009, 2011, 2013), the HIV Drug Therapy Glasgow Meeting (years 2008, 2010, 2012, 2014), and the International Conference on HIV Treatment and Prevention Adherence (years 2010–2014).

Eligible studies met the following criteria: (1) randomized controlled (cross-over) trial (2) comparing monitoring-informed counseling as one of the intervention components versus not receiving such counseling. Intervention groups could thus consist of multi-component and single component interventions; (3) outcomes are medication adherence and/or viral load concentrations; 4) participants are HIV-infected persons prescribed cART for a chronic HIV infection. We included English language papers only.

### Data extraction

We extracted the following information from each study: name of the first author, year of publication, sample size, whether patients were initiating, restarting, or switching a cART regimen or were already on ART, whether the intervention was only administered to patients with low pre-intervention adherence levels (yes/no), or if patients were triaged to different levels of intervention intensity depending on their adherence level (yes/no), the percentage of patients with undetectable viral loads at baseline, duration of the intervention period (weeks), number of intervention sessions, and intervention components. The categorization of intervention components was adapted from two previous systematic reviews of anti-retroviral adherence interventions (6, 8). Intervention components additional to (1) monitoring-informed counseling were coded as: (2) didactic provision of information about HIV, cART, and adherence, (3) behavioral, cognitive behavioral, or motivational counseling, (4) provision of reminder devices, (5) social support enlistment, (6) depression screening, treatment, or referral, (7) financial incentives for good adherence, and (8) substance use screening, treatment, or referral. We calculated the number of intervention components per study. Both authors independently extracted information, and discrepancies were resolved through discussion. When more than one type of intervention was tested, data from each arm of the intervention were considered as separate data points.

### Statistical analysis

We defined adherence as the percentage of prescribed doses of cART taken. We used the standardized mean difference (SMD) as the common effect size to express the difference in adherence between intervention and control groups. If studies did not provide the SMD, we contacted authors to get additional data. If no additional data were available, we calculated the SMD from correlation coefficients, means and SDs, odds ratios (OR), *t*-, *x*^2^-, or *F*-statistics, contingency table data, or exact *p* values (14). When studies reported an insignificant effect on adherence without data, we assigned a value to the SMD of 0.01. Values of the SMD of 0.2–0.49, 0.5–0.79, and ≥0.8 can be interpreted as small, medium, and large effects, respectively (15).

We used the OR as the common effect size to express the difference in the percentage of patients with an undetectable viral load in intervention versus control groups. When studies reported an insignificant effect of the intervention on viral load without data, we assumed an OR of 1.01. Random-effect models with inverse variance weights were used to aggregate individual SMDs and ORs into pooled effect estimates with 95% confidence limits (CI) using Review Manager 5.3.

We compared pooled effect estimates of adherence and undetectable viral loads between studies in which monitoring-informed counseling was the only intervention component with studies in which monitoring-informed counseling was one out of multiple intervention components. We conducted a sensitivity analysis to examine potential bias resulting from over-representation of studies with more than one intervention arm. We examined the extent to which results would change when studies with more than one intervention arm were excluded from the analysis or if only a single intervention arm was included.

We examined whether variation in effect sizes of adherence and viral load were significantly associated with study design features. We investigated the effect of the following study design features: whether patients were initiating, restarting, or switching a cART regimen (yes/no) or were already on ART, the percentage of patients with undetectable viral loads at baseline, whether the intervention was only administered to patients with low pre-intervention adherence levels (yes/no), or if patients were triaged to different levels of intervention intensity based on their adherence level (yes/no), duration of the intervention period (weeks), number of intervention contacts, whether intervention components administered to the intervention group included the following: didactic provision of information about HIV, cART, and adherence (yes/no), behavioral, cognitive behavioral, or motivational counseling (yes/no), provision of reminder devices (yes/no), social support enlistment (yes/no), depression screening/treatment/referral (yes/no), financial incentives for good adherence (yes/no), and substance use screening/treatment/referral (yes/no).

Subgroup analysis were performed by grouping effect sizes for adherence and viral load by study design feature and assessing heterogeneity between groups using the between-group *Q* statistic (*Q*-between) within a mixed effects model using the method of moments estimation. These analyses were conducted using the SPSS macro’s MetaF and MetaReg from Lipsey and Wilson (14, 16). We performed meta-regression analysis with method of moment estimation to assess the relationship of the number of intervention components per study with the SMD in adherence and the log OR of undetectable HIV RNA using Comprehensive Meta-Analysis version 2. We examined the presence of publication bias by the visual inspection of funnel plot symmetry and formally with Egger’s regression intercept.

## Results

**Figure 1:**
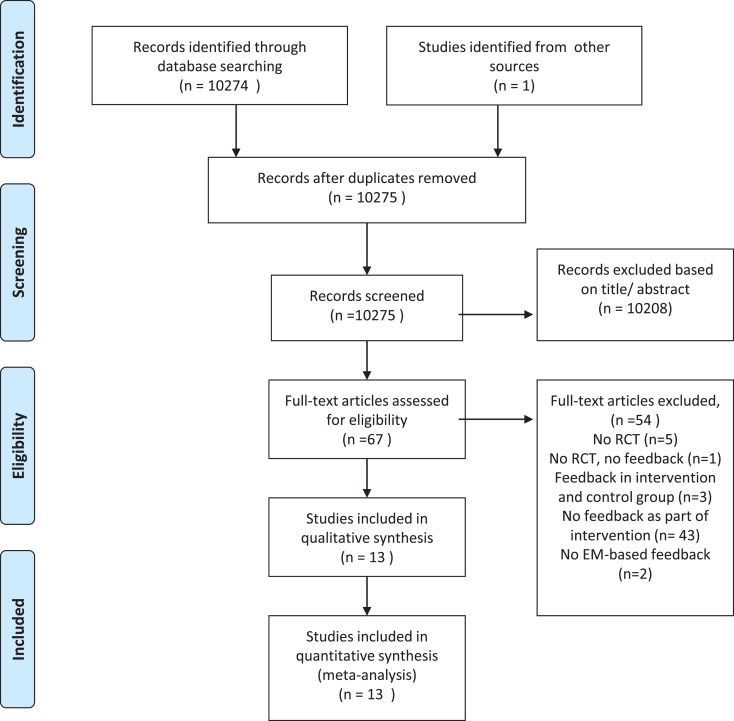
**Flow diagram**.

Our literature search yielded a total of 10,274 potentially relevant articles. We found an additional article from another data source, resulting in a total of 10,275 potentially relevant articles. All articles were subsequently screened on the title and abstract. After reading the full text of 67 articles, we excluded 54 articles mainly because the intervention did not consist of monitoring-informed counseling. Thus, a total of 13 articles, reporting on 1419 patients, were found to meet inclusion criteria and were entered in our meta-analysis (Figure 1). Characteristics of the included studies are shown in Table 1. We contacted three authors, but could not get any additional data.

**Table 1:** **Characteristics of included studies**.

Study	N	Starting/switching cART	Low pre-intervention adherence	% with undetectable baseline VL	Intervention components	Intervention duration (weeks)	Number of intervention sessions
Davies et al (17)	145	No	No	–	1	48	–
de Bruin et al (12)*	133	No	No**	84	1, 2, 3	36	4
Engelbrecht (18)	88	No	No	69	1, 4	16	4
Gross et al (19)*	180	Yes	No	0	1, 2, 3, 5, 6, 8	52	22
Koenig et al (20,21)	139	Yes	No	0	1, 2, 3, 4, 5, 6, 8	24	11
Rigsby et al (22)*	55	No	No	35	1, 3	4	5
					1, 3, 7	
Rosen et al (23)*	56	No	Yes	57	1, 3, 7, 8	16	16
Sabin et al (24)*	64	No	No**	88	1	24	6
Sabin et al (25)*	116	No	No**	99	1, 4	24	6
Safren et al (26)*	45	No	No	–	1, 2, 3, 4, 6	48	10–12
Smith et al (27)*	43	Yes	No	0	1, 2, 3, 5	12	4
Wagner et al (28)*	199	Yes	No	15	1, 3	48	5
Wilson et al 2010 (13)	156	No	No	0	1	−	2

(1) monitoring-informed counseling (2) didactic provision of information about HIV, cART and adherence; (3) behavioral, cognitive behavioral, or motivational counseling; (4) provision of reminder device; (5) social support enlistment; (6) depression screening/treatment/referral; (7) financial incentives for good adherence; (8) substance use screening/treatment/referral. *The study had reported significant improvement in adherence due to the intervention of interest. **Patients were triaged to different levels of intervention intensity depending on their level of adherence.

**Figure 2:**
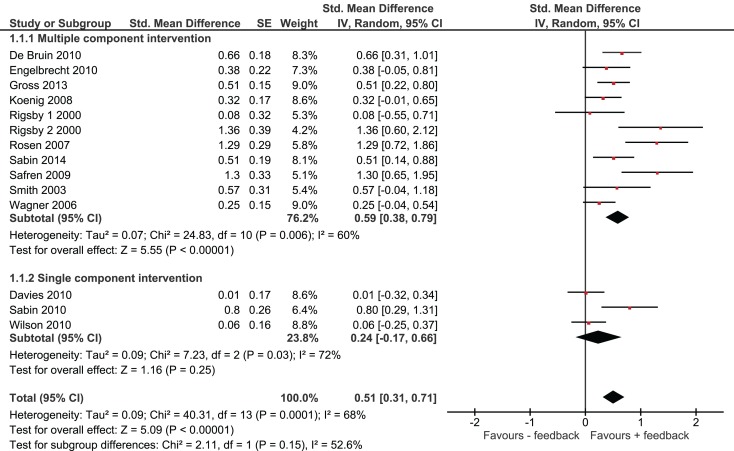
**Effect of interventions on adherence**.

**Figure 3:**
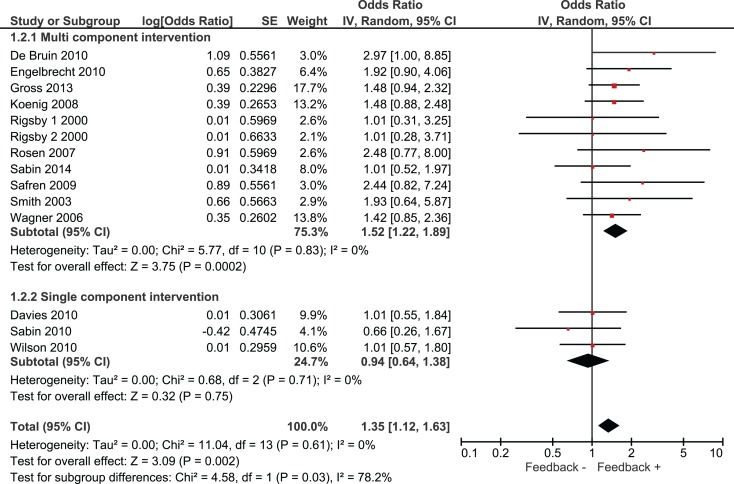
**Effect of interventions on the likelihood of undetectable HIV RNA**.

Overall, we found that adherence was significantly higher in intervention groups, which had received monitoring-informed counseling as part of the intervention compared with control groups that did not receive such counseling (SMD 0.51, 95% CI 0.31–0.71) (Figure 2). This represents an improvement of a moderate magnitude in terms of effect sizes. Moreover, patients in these intervention groups were more likely to have undetectable HIV RNA concentrations than patients in control groups that did not receive such counseling (OR 1.35, 95% CI 1.12–1.63) (Figure 3).

We identified three studies in which monitoring-informed counseling was the single intervention component and was compared with a control group not receiving such counseling (13, 14, 22). In these three studies, the effect of the intervention on adherence (SMD 0.24, 95% CI −0.17 to 0.66) (Figure 2) and on the likelihood of undetectable HIV RNA concentrations (OR 0.94, 95% CI 0.64–1.38) (Figure 3) was not statistically significant.

A total of 12 out 13 studies included compared a single intervention arm with a control group. In one study, two intervention arms were compared with the same control group (20). One of the intervention arms of this study provided financial incentives for good adherence in addition to the other intervention components, whereas the other intervention arm did not provide financial incentives. Excluding this study from our analysis or including only the intervention arm without financial incentive resulted in pooled effect estimates for adherence of SMD: 0.49 (95% CI 0.30–0.69) and SMD: 0.47 (95% CI 0.28–0.66), respectively, and for the likelihood of achieving undetectable viral load of OR: 1.37 (95% CI 1.13–1.66) and OR: 1.36 (95% CI 1.12–1.64), respectively.

Variation in effect sizes of adherence were significantly associated with the study design features; percentage of patients with undetectable viral load at baseline and providing financial incentives for good adherence. Studies with a lower percentage of patients with undetectable viral loads at baseline (dichotomized at the median of 35%) yielded larger effect sizes than studies with a higher percentage of patients with undetectable viral loads at baseline (SMD 0.65 versus 0.31, *Q* = 6.87, *p* = 0.009). Studies providing financial incentives for good adherence (22, 23) yielded larger effect sizes than studies without financial incentives (SMD 1.32 versus 0.40, *Q* = 10.83, *p* = 0.001). Both effects remained statistically significant in a multivariate model.

Variation in effect sizes for undetectable viral loads were significantly associated with whether the intervention components included didactic provision of information about HIV, cART, and adherence, or behavioral, cognitive behavioral, or motivational counseling. Studies in which the intervention components included didactic provision of information about HIV, cART, and adherence yielded higher effect sizes than studies not including this intervention component (OR 1.81 versus OR 1.19, *Q* = 9.91, *p* = 0.0016). Studies in which the intervention components included behavioral, cognitive behavioral, or motivational counseling yielded higher effect sizes than studies not including this component (OR 1.64 versus OR 1.08, *Q* = 11.89, *p* = 0.0006). We were unable to include both variables in a multivariate model due to high collinearity.

**Figure 4:**
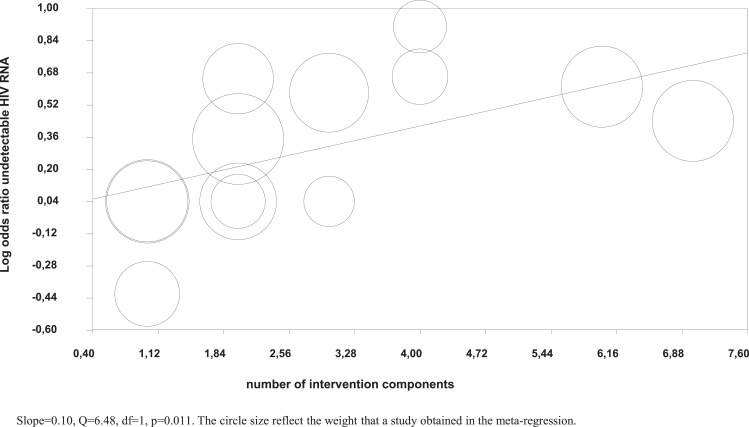
**Meta-regression of number of intervention components on the log odds of undetectable HIV RNA**.

Meta-regression analysis showed that a higher number of intervention components were significantly associated with a higher likelihood of undetectable HIV RNA (Figure 4), but not with higher adherence in the intervention groups.

**Figure 5:**
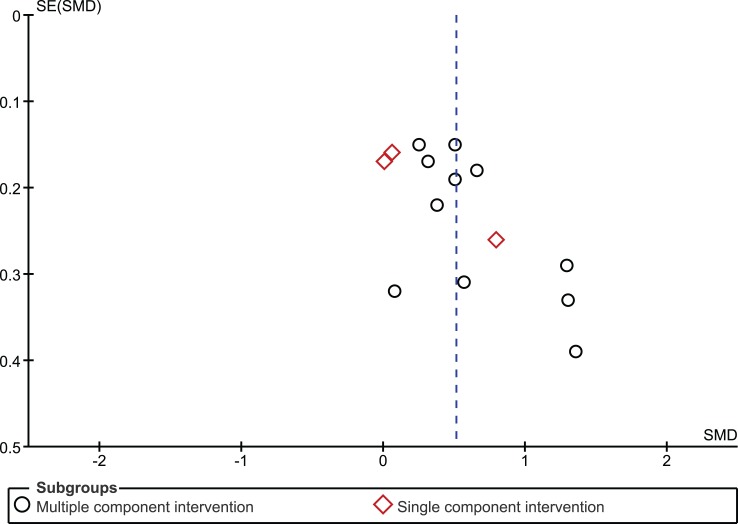
**Funnel plot medication adherence**.

**Figure 6:**
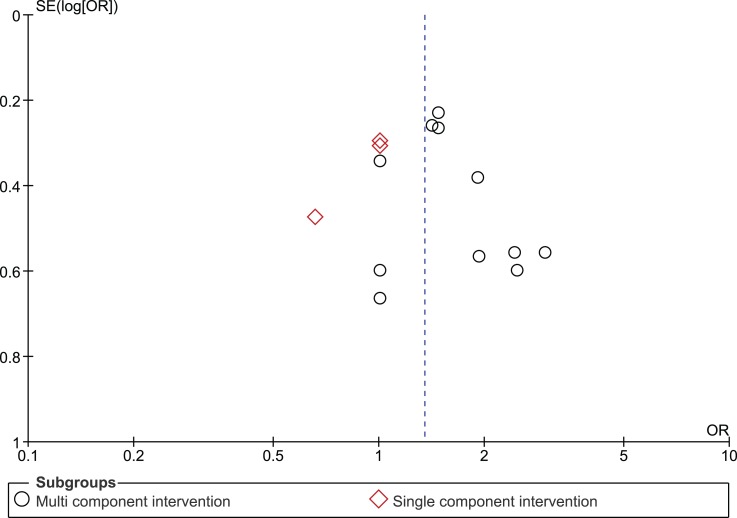
**Funnel plot undetectable HIV RNA**.

The funnel plot for the outcome measure medication adherence was suggestive of publication bias (Egger’s regression intercept *p* = 0.04), with an absence of small studies yielding negative effects (Figure 5). The funnel plot for the outcome measure virologic treatment response was not suggestive of publication bias (Egger’s regression intercept *p* = 0.99) (Figure 6).

## Discussion

Our meta-analysis of randomized controlled trials (RCTs) investigating the effect of monitoring-informed counseling on treatment adherence and virologic treatment response, yielded significantly improved adherence and virologic treatment response only when such counseling was part of a multi-component intervention. The improvement in adherence constituted a medium-sized effect. The improvement in medication adherence achieved in the intervention groups was clinically relevant as it was accompanied by an increased likelihood of having an undetectable viral load.

We distinguished between studies in which monitoring-informed counseling was the only intervention component and studies in which monitoring-informed counseling was one out of multiple intervention components. In the vast majority of studies included, monitoring-informed counseling was one out of multiple intervention components that patients received. Consequently, the separate contribution of monitoring-informed counseling to the improved levels of adherence and virologic treatment response is difficult to establish. However, this reflects the current state of the art in HIV adherence support research in which most interventions consist of multiple components (29). Moreover, it reflects the clinical reality that adherence is behavior that may be affected by multifactorial barriers that may best be addressed by comprehensive interventions (9). Multi-component interventions may increase the likelihood of having an impact on adherence and treatment outcomes compared with single component interventions. Researchers may first combine approaches to document an effect and in later studies attempt to isolate effects of intervention features.

We identified only three single component studies with divergent results. Two single component studies found insignificant effects of the intervention on adherence (13, 14). By contrast, one single component study found significantly improved adherence in the intervention group (22). In one of the single component studies with insignificant results, the lack of effect of the intervention was attributed to inadequate adherence counseling techniques of the health care providers who delivered the intervention (13). Although the amount of adherence-related dialog increased in the intervention group, little of that dialog was problem solving in nature but tended to have a scolding or lecturing quality (13).

By contrast, the single component study that yielded improved levels of adherence in the intervention group mentioned that participating health care providers had received practice and role-playing training sessions during which it had been emphasized that the goal of counseling was to help subjects to improve their medication-taking behavior, not to scold them about poor adherence (22). The authors speculated that for patients with adherence problems, monitoring-informed counseling offered an opportunity for meaningful discussion about medication-taking issues specific to the individual and point in time, which may have provided patients just the focused discussion of behavior changes that they needed (22). Given the quest for effective and practical interventions to promote medication adherence (30), it would be most interesting to see if future studies employing a similar relatively simple monitoring-informed counseling with prior training of health care providers would also result in improved adherence.

We aimed to identify study design features that were associated with larger effect sizes for adherence and virologic treatment response. Lower percentage of patients with undetectable viral loads at baseline and providing financial incentives for good adherence were significantly associated with larger effect sizes for adherence but not for virologic treatment response. The clinical relevance of these findings is therefore uncertain. Studies including the didactic provision of information about HIV, cART, and adherence, or behavioral, cognitive behavioral, or motivational counseling as intervention components yielded larger effect sizes for virologic treatment response than studies that did not include these intervention components. We were unable to assess the independent effect of these two intervention components in a multivariate model, due to the low number of included studies and due to the fact that many studies including one of the two intervention components also included the other component. While the multiple component studies were associated with larger improvements in adherence and virologic treatment response than the single component studies, it remains thus largely unknown which intervention components are responsible for this difference.

There is also more a fundamental reason why it could be difficult to determine which adherence intervention components are most responsible for improvements in adherence than others. It is increasingly recognized that multifactorial barriers may influence patient adherence and that these barriers may differ between patients and change within patients over time. There is also increasing recognition that interventions should be targeted to people who are clearly identified as needing that specific intervention. By analogy with a medical condition that can only be adequately treated when an accurate diagnosis is established, the treatment, i.e., adherence intervention, should be matched to the diagnosis, i.e., barriers to adherence that the patient is experiencing (31). For example, reminder devices are not that likely to help people whose adherence barriers are not related to problems remembering doses. Consequently, adherence intervention components that are highly effective for a particular patient may be largely ineffective for another patient depending on the specific problems with adherence that a particular patient is experiencing. The most effective interventions are probably those that carefully tailor the intervention components to the adherence problems that an individual patient is experiencing.

There has been attention in the field to the content of adherence care that is provided in control groups of adherence intervention studies. It was previously found that the difference in adherence intervention components provided in the intervention and control groups was a significant predictor of the difference in viral load and adherence success rates between intervention and control groups (32). In the present study, the difference in adherence intervention components provided in the intervention and control groups was neither a significant predictor of the difference in virologic success nor the difference in adherence between intervention and control groups (data not shown). This finding may have been due, however, to a limited description of adherence care provided to control groups in many of the included studies.

The present study has several limitations. First, because in the vast majority of studies monitoring-informed counseling was one out of multiple intervention components, the separate contribution of such counseling to the improved levels of adherence and virologic treatment response is difficult to establish.

Second, we searched a single electronic database only, i.e., PubMed, which may have resulted in publication bias. However, we supplemented this database with searches in abstracts of the most relevant HIV conferences for the subject of medication adherence.

Third, if studies reported insignificant differences in adherence or virologic response between intervention and control groups without data, we assumed an effect size of 0.01. This may have been a too conservative estimate, which may have resulted in an underestimate of the effectiveness of the concerning interventions.

Fourth, we categorized adherence intervention components in several broad categories, for example, behavioral, cognitive behavioral, or motivational counseling. Within these broad categories, distinct types of intervention components may have had a different impact on adherence and virologic response that will remain undetected in the present global analysis, e.g., cognitive behavioral counseling could have had a different impact on adherence and virologic response than motivational interviewing.

Fifth, when more than one type of intervention was tested within a single study, we considered data from each arm of the intervention as separate data points. This may have resulted in an over-representation of a study with more than one intervention arm. However, a sensitivity analysis in which we excluded this study from our analysis or included only one of the intervention arms did not change our overall results.

In conclusion, monitoring-informed counseling improved medication adherence and virologic response compared with control groups not receiving such counseling in studies in which it was one out of multiple intervention components, but not in studies where it was the only intervention component.

## Conflict of Interest Statement

The authors declare that the research was conducted in the absence of any commercial or financial relationships that could be construed as a potential conflict of interest.
