# Corrigendum: Electronic medication monitoring-informed counseling to improve adherence to combination anti-retroviral therapy and virologic treatment outcomes: a meta-analysis

**DOI:** 10.3389/fpubh.2016.00090

**Published:** 2016-05-11

**Authors:** Nienke Langebeek, Pythia Nieuwkerk

**Affiliations:** ^1^Department of Internal Medicine, Rijnstate Hospital, Arnhem, Netherlands; ^2^Department of Medical Psychology, Academic Medical Center, Amsterdam, Netherlands

**Keywords:** adherence, compliance, HIV infection, antiretroviral therapy, meta-analysis

An error occurred with citations in the Discussion section.

All citations of reference #22 (study from Rigsby et al.) on page 8, column 1, paragraph 2 and 3, should be replaced by citations of reference #24 (study from Sabin et al.).

The correction does not affect the scientific validity of the results.

## Author Contributions

All authors listed, have made substantial, direct and intellectual contribution to the work, and approved it for publication.

## Conflict of Interest Statement

The authors declare that the research was conducted in the absence of any commercial or financial relationships that could be construed as a potential conflict of interest.

